# Multi-ancestry genome-wide association study of gestational diabetes mellitus highlights genetic links with type 2 diabetes

**DOI:** 10.1093/hmg/ddac050

**Published:** 2022-02-26

**Authors:** Natalia Pervjakova, Gunn-Helen Moen, Maria-Carolina Borges, Teresa Ferreira, James P Cook, Catherine Allard, Robin N Beaumont, Mickaël Canouil, Gad Hatem, Anni Heiskala, Anni Joensuu, Ville Karhunen, Soo Heon Kwak, Frederick T J Lin, Jun Liu, Sheryl Rifas-Shiman, Claudia H Tam, Wing Hung Tam, Gudmar Thorleifsson, Toby Andrew, Juha Auvinen, Bishwajit Bhowmik, Amélie Bonnefond, Fabien Delahaye, Ayse Demirkan, Philippe Froguel, Kadri Haller-Kikkatalo, Hildur Hardardottir, Sandra Hummel, Akhtar Hussain, Eero Kajantie, Elina Keikkala, Amna Khamis, Jari Lahti, Tove Lekva, Sanna Mustaniemi, Christine Sommer, Aili Tagoma, Evangelia Tzala, Raivo Uibo, Marja Vääräsmäki, Pia M Villa, Kåre I Birkeland, Luigi Bouchard, Cornelia M Duijn, Sarah Finer, Leif Groop, Esa Hämäläinen, Geoffrey M Hayes, Graham A Hitman, Hak C Jang, Marjo-Riitta Järvelin, Anne Karen Jenum, Hannele Laivuori, Ronald C Ma, Olle Melander, Emily Oken, Kyong Soo Park, Patrice Perron, Rashmi B Prasad, Elisabeth Qvigstad, Sylvain Sebert, Kari Stefansson, Valgerdur Steinthorsdottir, Tiinamaija Tuomi, Marie-France Hivert, Paul W Franks, Mark I McCarthy, Cecilia M Lindgren, Rachel M Freathy, Deborah A Lawlor, Andrew P Morris, Reedik Mägi

**Affiliations:** Estonian Genome Centre, Institute of Genomics, University of Tartu, Tartu 51010, Estonia; Institute of Clinical Medicine, Faculty of Medicine, University of Oslo, Oslo, Norway; Diamantina Institute, The University of Queensland, Woolloongabba QLD 4102, Australia; K.G. Jebsen Center for Genetic Epidemiology, Department of Public Health and Nursing, NTNU, Norwegian University of Science and Technology, Trondheim, Norway; Population Health Science, Bristol Medical School, University of Bristol, Bristol, UK; Population Health Science, Bristol Medical School, University of Bristol, Bristol, UK; MRC Integrative Epidemiology Unit, University of Bristol, Bristol, UK; Big Data Institute, Li Ka Shing Center for Health for Health Information and Discovery, Oxford University, Oxford, UK; Department of Health Data Science, University of Liverpool, Liverpool, UK; Centre de Recherche du Centre Hospitalier Universitaire de Sherbrooke (CRCHUS), Universite de Sherbrooke, Quebec, Canada; Institute of Biomedical and Clinical Science, College of Medicine and Health, University of Exeter, Exeter, UK; Inserm U1283, CNRS UMR 8199, European Genomic Institute for Diabetes, Institut Pasteur de Lille, Lille F-59000, France; University of Lille, Lille University Hospital, Lille F-59000, France; Department of Clinical Sciences, Lund University, Skåne University Hospital, Lund University Diabetes Centre, Malmö SE-20502, Sweden; Centre for Life-Course Health Research, Faculty of Medicine, University of Oulu, Oulu, Finland; Department of Public Health Solutions, Finnish Institute for Health and Welfare, Helsinki, Finland; Research Program for Clinical and Molecular Metabolism, Faculty of Medicine, University of Helsinki, Helsinki, Finland; Centre for Life-Course Health Research, Faculty of Medicine, University of Oulu, Oulu, Finland; School of Public Health, Department of Epidemiology and Biostatistics, Imperial College London, St Mary's Hospital, London, UK; Department of Internal Medicine, Seoul National University Hospital, Seoul, Republic of Korea; Division of Endocrinology, Metabolism, and Molecular Medicine, Department of Medicine, Northwestern University Feinberg School of Medicine, Chicago, IL 60611, USA; Department of Epidemiology, Erasmus Medical Center, Rotterdam, the Netherlands; Nuffield Department of Population Health, University of Oxford, Oxford, UK; Department of Population Medicine, Harvard Medical School and Harvard Pilgrim Health Care Institute, Boston, MA, USA; Department of Medicine and Therapeutics, The Chinese University of Hong Kong, Hong Kong SAR, The People's Republic of China; Department of Obstetrics and Gynaecology, The Chinese University of Hong Kong, Hong Kong SAR, The People's Republic of China; deCODE Genetics/Amgen, Inc., Reykjavik, Iceland; Inserm U1283, CNRS UMR 8199, European Genomic Institute for Diabetes, Institut Pasteur de Lille, Lille F-59000, France; University of Lille, Lille University Hospital, Lille F-59000, France; Department of Metabolism, Digestion and Reproduction, Imperial College London, London, UK; Centre for Life-Course Health Research, Faculty of Medicine, University of Oulu, Oulu, Finland; Centre of Global Health Research, Diabetic Association of Bangladesh, Dhaka, Bangladesh; Inserm U1283, CNRS UMR 8199, European Genomic Institute for Diabetes, Institut Pasteur de Lille, Lille F-59000, France; University of Lille, Lille University Hospital, Lille F-59000, France; Department of Metabolism, Digestion and Reproduction, Imperial College London, London, UK; Inserm U1283, CNRS UMR 8199, European Genomic Institute for Diabetes, Institut Pasteur de Lille, Lille F-59000, France; University of Lille, Lille University Hospital, Lille F-59000, France; Department of Epidemiology, Erasmus Medical Center, Rotterdam, the Netherlands; Section of Statistical Multi-omics, Department of Clinical and Experimental Research, University of Surrey, Surrey, UK; Inserm U1283, CNRS UMR 8199, European Genomic Institute for Diabetes, Institut Pasteur de Lille, Lille F-59000, France; University of Lille, Lille University Hospital, Lille F-59000, France; Department of Metabolism, Digestion and Reproduction, Imperial College London, London, UK; Department of Immunology, Institute of Biomedicine and Translational Medicine, University of Tartu, Tartu, Estonia; Faculty of Medicine, University of Iceland, Reykjavik, Iceland; Livio Reykjavik, Reykjavik, Iceland; Institute of Diabetes Research, Helmholtz Zentrum München, German Research Center for Environmental Health, Munich-Neuherberg, Germany; Forschergruppe Diabetes, Technical University Munich, at Klinikum rechts der Isar, Munich, Germany; Centre of Global Health Research, Diabetic Association of Bangladesh, Dhaka, Bangladesh; Faculty of Health Sciences, Nord University, Bodø, Norway; Population Health Unit, Finnish Institute for Health and Welfare, Helsinki and Oulu, Finland; PEDEGO Research Unit, MRC Oulu, Oulu University Hospital and University of Oulu, Oulu, Finland; Department of Clinical and Molecular Medicine, Norwegian University of Science and Technology, Trondheim, Norway; Population Health Unit, Finnish Institute for Health and Welfare, Helsinki and Oulu, Finland; PEDEGO Research Unit, MRC Oulu, Oulu University Hospital and University of Oulu, Oulu, Finland; Inserm U1283, CNRS UMR 8199, European Genomic Institute for Diabetes, Institut Pasteur de Lille, Lille F-59000, France; University of Lille, Lille University Hospital, Lille F-59000, France; Department of Metabolism, Digestion and Reproduction, Imperial College London, London, UK; Department of Psychology and Logopedics, University of Helsinki, Helsinki, Finland; Research Institute of Internal Medicine, Oslo University Hospital, Oslo, Norway; Population Health Unit, Finnish Institute for Health and Welfare, Helsinki and Oulu, Finland; PEDEGO Research Unit, MRC Oulu, Oulu University Hospital and University of Oulu, Oulu, Finland; Department of Endocrinology, Morbid Obesity and Preventive Medicine, Oslo University Hospital, Oslo, Norway; Department of Immunology, Institute of Biomedicine and Translational Medicine, University of Tartu, Tartu, Estonia; School of Public Health, Department of Epidemiology and Biostatistics, Imperial College London, St Mary's Hospital, London, UK; Department of Immunology, Institute of Biomedicine and Translational Medicine, University of Tartu, Tartu, Estonia; PEDEGO Research Unit, MRC Oulu, Oulu University Hospital and University of Oulu, Oulu, Finland; Population Health Unit, Finnish Institute for Health and Welfare, Helsinki and Oulu, Finland; Department of Obstetrics and Gynaecology, University of Helsinki and Helsinki University Hospital, Helsinki, Finland; Hyvinkää Hospital, Helsinki and Uusimaa Hospital District, Hyvinkää, Finland; Institute of Clinical Medicine, Faculty of Medicine, University of Oslo, Oslo, Norway; Department of Transplantation Medicine, Oslo University Hospital, Oslo, Norway; Department of Biochemistry and Functional Genomics, Faculty of Medicine and Health Sciences, Universite de Sherbrooke, Quebec, Canada; Department of Medical Biology, Centre Intégré Universitaire de Santé et de Services Sociaux du Saguenay–Lac-St-Jean – Hôpital de Chicoutimi, Québec, Canada; Department of Epidemiology, Erasmus Medical Center, Rotterdam, the Netherlands; Nuffield Department of Population Health, University of Oxford, Oxford, UK; Centre for Genomics and Child Health, Blizard Institute, Barts and the London School of Medicine and Dentistry, Queen Mary University of London, London, UK; Department of Clinical Sciences, Lund University, Skåne University Hospital, Lund University Diabetes Centre, Malmö SE-20502, Sweden; Institute for Molecular Medicine Finland (FIMM), Helsinki Institute of Life Science, University of Helsinki, Helsinki, Finland; Department of Clinical Chemistry, University of Eastern Finland, Kuopio, Finland; Division of Endocrinology, Metabolism, and Molecular Medicine, Department of Medicine, Northwestern University Feinberg School of Medicine, Chicago, IL 60611, USA; Center for Genetic Medicine, Northwestern University Feinberg School of Medicine, Chicago, IL 60611, USA; Department of Anthropology, Northwestern University, Evanston, IL 60208, USA; Centre for Genomics and Child Health, Blizard Institute, Barts and the London School of Medicine and Dentistry, Queen Mary University of London, London, UK; Department of Internal Medicine, Seoul National University Bundang Hospital, Seongnam, Republic of Korea; Department of Internal Medicine, Seoul National University College of Medicine, Seoul, Republic of Korea; Centre for Life-Course Health Research, Faculty of Medicine, University of Oulu, Oulu, Finland; School of Public Health, Department of Epidemiology and Biostatistics, Imperial College London, St Mary's Hospital, London, UK; General Practice Research Unit (AFE), Department of General Practice, Institute of Health and Society, Faculty of Medicine, University of Oslo, Post Box 1130 Blindern, Oslo 0318, Norway; Institute for Molecular Medicine Finland (FIMM), Helsinki Institute of Life Science, University of Helsinki, Helsinki, Finland; Department of Obstetrics and Gynecology, Tampere University, Hospital and Faculty of Medicine and Health Technology, Center for Child, Adolescent, and Maternal Health, Tampere University, Tampere, Finland; Medical and Clinical Genetics, University of Helsinki and Helsinki University Hospital, Helsinki, Finland; Department of Medicine and Therapeutics, The Chinese University of Hong Kong, Hong Kong SAR, The People's Republic of China; Laboratory for Molecular Epidemiology in Diabetes, Li Ka Shing Institute of Health Sciences, The Chinese University of Hong Kong, Hong Kong SAR, The People's Republic of China; Hong Kong Institute of Diabetes and Obesity, The Chinese University of Hong Kong, Hong Kong SAR, The People's Republic of China; Department of Clinical Sciences, Lund University, Skåne University Hospital, Lund University Diabetes Centre, Malmö SE-20502, Sweden; Department of Population Medicine, Harvard Medical School and Harvard Pilgrim Health Care Institute, Boston, MA, USA; Department of Internal Medicine, Seoul National University Hospital, Seoul, Republic of Korea; Department of Internal Medicine, Seoul National University College of Medicine, Seoul, Republic of Korea; Department of Molecular Medicine and Biopharmaceutical Sciences, Graduate School of Convergence Science and Technology, Seoul National University, Seoul, Republic of Korea; Centre de Recherche du Centre Hospitalier Universitaire de Sherbrooke (CRCHUS), Universite de Sherbrooke, Quebec, Canada; Department of Medicine, Faculty of Medicine and Health Sciences, University of Sherbrook, Québec, Canada; Department of Clinical Sciences, Lund University, Skåne University Hospital, Lund University Diabetes Centre, Malmö SE-20502, Sweden; Department of Endocrinology, Morbid Obesity and Preventive Medicine, Oslo University Hospital, Oslo, Norway; Institute of Clinical Medicine, Faculty of Medicine, University of Oslo, Oslo, Norway; Centre for Life-Course Health Research, Faculty of Medicine, University of Oulu, Oulu, Finland; deCODE Genetics/Amgen, Inc., Reykjavik, Iceland; Faculty of Medicine, University of Iceland, Reykjavik, Iceland; deCODE Genetics/Amgen, Inc., Reykjavik, Iceland; Institute for Molecular Medicine Finland (FIMM), Helsinki Institute of Life Science, University of Helsinki, Helsinki, Finland; Department of Clinical Sciences, Lund University, Skåne University Hospital, Lund University Diabetes Centre, Malmö SE-20502, Sweden; Department of Endocrinology, Abdominal Centre, Helsinki University Hospital, Helsinki, Finland; Folkhalsan Research Center, Helsinki, Finland; Department of Population Medicine, Harvard Medical School and Harvard Pilgrim Health Care Institute, Boston, MA, USA; Department of Medicine, Faculty of Medicine and Health Sciences, University of Sherbrook, Québec, Canada; Diabetes Unit, Massachusetts General Hospital, Boston, MA, USA; Department of Clinical Sciences, Lund University, Malmö, Sweden; Department of Nutrition, Harvard School of Public Health, Boston, MA, USA; Oxford Centre for Diabetes, Endocrinology and Metabolism, Radcliffe Department of Medicine, University of Oxford, Oxford, UK; Wellcome Centre for Human Genetics, Nuffield Department of Medicine, University of Oxford, Oxford, UK; Big Data Institute, Li Ka Shing Center for Health for Health Information and Discovery, Oxford University, Oxford, UK; Wellcome Centre for Human Genetics, Nuffield Department of Medicine, University of Oxford, Oxford, UK; Program in Medical and Population Genetics, Broad Institute, Boston, MA, USA; Institute of Biomedical and Clinical Science, College of Medicine and Health, University of Exeter, Exeter, UK; Population Health Science, Bristol Medical School, University of Bristol, Bristol, UK; MRC Integrative Epidemiology Unit, University of Bristol, Bristol, UK; Bristol NIHR Biomedical Research Centre, Bristol, UK; Centre for Genetics and Genomics Versus Arthritis, Centre for Musculoskeletal Research, Division of Musculoskeletal and Dermatological Sciences, University of Manchester, Manchester, UK; Estonian Genome Centre, Institute of Genomics, University of Tartu, Tartu 51010, Estonia

## Abstract

Gestational diabetes mellitus (GDM) is associated with increased risk of pregnancy complications and adverse perinatal outcomes. GDM often reoccurs and is associated with increased risk of subsequent diagnosis of type 2 diabetes (T2D). To improve our understanding of the aetiological factors and molecular processes driving the occurrence of GDM, including the extent to which these overlap with T2D pathophysiology, the GENetics of Diabetes In Pregnancy Consortium assembled genome-wide association studies of diverse ancestry in a total of 5485 women with GDM and 347 856 without GDM. Through multi-ancestry meta-analysis, we identified five loci with genome-wide significant association (*P* < 5 × 10^−8^) with GDM, mapping to/near *MTNR1B* (*P* = 4.3 × 10^−54^), *TCF7L2* (*P* = 4.0 × 10^−16^), *CDKAL1* (*P* = 1.6 × 10^−14^), *CDKN2A-CDKN2B* (*P* = 4.1 × 10^−9^) and *HKDC1* (*P* = 2.9 × 10^−8^). Multiple lines of evidence pointed to the shared pathophysiology of GDM and T2D: (i) four of the five GDM loci (not *HKDC1*) have been previously reported at genome-wide significance for T2D; (ii) significant enrichment for associations with GDM at previously reported T2D loci; (iii) strong genetic correlation between GDM and T2D and (iv) enrichment of GDM associations mapping to genomic annotations in diabetes-relevant tissues and transcription factor binding sites. Mendelian randomization analyses demonstrated significant causal association (5% false discovery rate) of higher body mass index on increased GDM risk. Our results provide support for the hypothesis that GDM and T2D are part of the same underlying pathology but that, as exemplified by the *HKDC1* locus, there are genetic determinants of GDM that are specific to glucose regulation in pregnancy.

## Introduction

Gestational diabetes mellitus (GDM), defined as hyperglycaemia with onset or first recognition during pregnancy, is associated with increased risk of pregnancy complications and adverse perinatal outcomes, including pre-eclampsia, stillbirth, large for gestational age, neonatal hypoglycaemia, preterm birth, low Apgar scores and admission to neonatal intensive care (1–4). Whilst hyperglycaemia commonly resolves postpartum, GDM often reoccurs (5) and is associated with subsequent diagnosis of type 2 diabetes (T2D) and coronary heart disease (6,7). Although the global prevalence of GDM is increasing, it varies according to population characteristics (such as maternal age, ancestry and obesity rates) and the criteria used for screening and diagnosis (8).

GDM and T2D share both genetic and non-genetic risk factors, including obesity, poor diet and sedentary lifestyle (9,10). Family studies have demonstrated that women with GDM have 30.1% probability of having at least one parent with T2D, compared to just 13.2% for pregnant women with normal glucose tolerance (11). Furthermore, women with a history of GDM appear to have a nearly 10-fold higher risk of developing T2D than those with a normoglycaemic pregnancy (7). Taken together, these observations support the hypothesis that the two diseases are part of the same underlying pathology, with pregnancy potentially acting as a stress test that reveals women at increased risk of GDM and/or T2D (12,13).

There have been considerable advances in our understanding of the genetic contribution to T2D through large-scale genome-wide association studies (GWAS) across diverse populations (14–17). In contrast, despite the observed familial clustering of GDM (18), most genetic association studies of the disease have focussed on evaluating the impact of previously reported loci for T2D and glycaemic traits in modest sample sizes (19). The most comprehensive systematic review of genetic susceptibility to GDM (from 23 studies) revealed association with T2D risk variants from seven loci, of which six are related to insulin secretion and one to insulin resistance (20). A genetic risk score (GRS) of risk variants across 34 loci associated with T2D and/or fasting glucose was significantly associated with GDM and improved predictive power over a model including only clinical variables (21). Variants associated with both insulin secretion and insulin resistance have also been used to construct an aggregated GRS that was shown to predict GDM risk, with and without adjustment for body mass index (BMI), maternal age and gestational age, although this score was not compared with established clinical predictors (22). To date, the largest GWAS of GDM has been undertaken in women from a Korean population, including 468 cases and 1242 non-diabetic controls in the discovery stage, with an additional 931 cases and 783 non-diabetic controls in the follow-up stage (23). Two loci were associated with GDM at genome-wide significance (*P* < 5 × 10^−8^), mapping near *MTNR1B* and *CDKAL1*, both of which have also been previously implicated in T2D risk.

To gain novel insight into the genetic architecture of GDM, the GENetics of Diabetes In Pregnancy (GenDIP) Consortium assembled GWAS of diverse ancestry in a total of 5485 women with GDM and 347 856 women without GDM. With these resources, we aimed to improve our understanding of the aetiological factors and molecular processes driving the occurrence of GDM, including the extent to which these overlap with T2D pathophysiology, and investigate the effects of potential causal metabolic risk factors on the disease through Mendelian randomization (MR).

## Results

We began by aggregating GDM association summary statistics across GWAS through multi-ancestry meta-analysis: the effective sample size was 72.2% European, 13.4% East Asian, 9.9% South Asian, 2.8% Hispanic/Latino and 1.7% African (Supplementary Material, Tables S1 and S2). To maximize sample size, we used a phenotype definition that makes best use of the information available in each study, including data from health records, oral glucose tolerance tests and self-report (Supplementary Material, Table S1). Each GWAS was imputed to reference panels from the 1000 Genomes Project (24), Haplotype Reference Consortium (25) or population-specific whole-genome sequence data (Supplementary Material, Table S3). Within each GWAS, GDM association summary statistics were derived for all single nucleotide variants (SNVs) passing quality control after appropriate adjustment to account for population structure (Supplementary Material, Table S3).

The most powerful methods for multi-ancestry meta-analysis allow for potential allelic effect heterogeneity on disease between population groups that cannot be accommodated in a fixed-effects model (26). Our primary analysis used MR-MEGA (27), which models heterogeneity between GWAS by including axes of genetic variation that represent ancestry as covariates in a meta-regression model. We considered three axes of genetic variation that separated the five ancestry groups, but which also revealed finer-scale genetic differences between GWAS of the same ancestry (Supplementary Material, Fig. S1). We also conducted multi-ancestry and ancestry-specific fixed-effects meta-analyses. We identified five loci at genome-wide significance in the multi-ancestry meta-regression (Table 1, Supplementary Material, Figs S2 and S3), including the previously reported associations from GDM GWAS at *MTNR1B* (rs10830963, *P* = 4.3 × 10^−54^) and *CDKAL1* (rs9348441, *P* = 1.6 × 10^−14^). The remaining three loci for GDM mapped to/near *TCF7L2* (rs7903146, *P* = 4.0 × 10^−16^), *CDKN2A-CDKN2B* (rs10811662, *P* = 4.1 × 10^−9^) and *HKDC1* (rs9663238, *P* = 2.9 × 10^−8^). Through approximate conditional analyses, conducted using ancestry-matched linkage disequilibrium (LD) reference panels for each GWAS, we observed no evidence for multiple distinct association signals at genome-wide significance at any of the five GDM loci (Supplementary Material, Fig. S4).

**Table 1:** Loci attaining genome-wide significant (*P* < 5 × 10^−8^) evidence of association with GDM in multi-ancestry meta-regression (MR-MEGA) of 5485 cases and 347 856 controls

**Locus**	**Lead SNV**	**Chr**	**Position (bp, b37)**	**Alleles**		**MR-MEGA *P*-value**	**Fixed-effects OR (95% CI)**
				**Risk**	**Other**		
*MTNR1B*	rs10830963	11	92 708 710	G	C	4.3 × 10^−54^	1.41 (1.35–1.47)
*TCF7L2*	rs7903146	10	114 758 349	T	C	4.0 × 10^−16^	1.22 (1.16–1.27)
*CDKAL1*	rs9348441	6	20 680 678	A	T	1.6 × 10^−14^	1.13 (1.08–1.18)
*CDKN2A-CDKN2B*	rs10811662	9	22 134 253	G	A	4.1 × 10^−9^	1.14 (1.09–1.20)
*HKDC1*	rs9663238	10	70 983 629	G	A	2.9 × 10^−8^	1.14 (1.09–1.19)

Chr: chromosome. OR: odds-ratio. CI: confidence interval.

We next sought to investigate the impact of differences in ancestry and phenotype definition between GWAS on heterogeneity in allelic effects at GDM loci. To do this, we extended the MR-MEGA meta-regression model to include an additional covariate to represent whether GDM status in the study was confirmed via ‘a universal blood-based test’ (Supplementary Material, Table S1). Here, we use this term to refer to a blood-based test that was applied to all participants, including a diagnostic oral glucose tolerance test (OGTT) or a screening glucose challenge or fasting glucose test, in contrast to clinician decision, risk factor screening or a lack of clarity on what basis women did or did not have a diagnostic OGTT. This model enables partitioning of heterogeneity into three components (Table 2). The first component captures heterogeneity that is correlated with genetic ancestry (that can be explained by the three axes of genetic variation), which can occur because of differences in the structure of LD between ancestry groups or interactions with lifestyle factors that vary across populations. The second component measures heterogeneity that can be explained by the use of a universal blood-based test to screen for or diagnose GDM. The final component reflects residual heterogeneity due to study design that cannot be explained by the first two components. The greatest evidence of ancestry-correlated heterogeneity (after accounting for the use of a universal blood-based test) was observed at the *CDKAL1* locus (*P*_HET_ = 3.4 × 10^−5^), where the lead SNV demonstrated stronger effects on GDM in GWAS of East Asian ancestry than in other populations, despite the risk allele being common in all ancestry groups (Supplementary Material, Fig. S5, Table S4). A similar pattern of ancestry-correlated heterogeneity in allelic effects on T2D has been reported at the *CDKAL1* locus (16). Weaker evidence of ancestry-correlated heterogeneity was observed at the *CDKN2A-CDKN2B* locus (*P*_HET_ = 0.0022), where there were marked differences in the effects on GDM of the lead SNV between GWAS undertaken in different ancestry groups (Supplementary Material, Fig. S5, Table S4). In contrast, there was no evidence of heterogeneity due to phenotype definition for any lead SNV, suggesting that differences in allelic effects between GWAS are more likely due to factors related to genetic ancestry than the use of a blood-based test in all women to screen for or diagnose GDM.

**Table 2:** Source of heterogeneity in allelic effects on GDM between GWAS for lead SNVs derived from meta-regression of 5485 cases and 347 856 controls

**Locus**	**Lead SNV**	**Heterogeneity source (*P*-value)**		
		**Ancestry**	**Universal blood-based test**	**Residual**
*MTNR1B*	rs10830963	0.14	0.41	0.67
*TCF7L2*	rs7903146	0.25	0.83	0.089
*CDKAL1*	rs9348441	3.4 × 10^−5^	0.28	0.15
*CDKN2A-CDKN2B*	rs10811662	0.0022	0.45	0.26
*HKDC1*	rs9663238	0.19	0.33	5.4 × 10^−5^

Of the five GDM loci identified at genome-wide significance in the trans-ancestry meta-regression, four have been previously implicated in T2D susceptibility: *MTNR1B*, *TCF7L2*, *CDKAL1* and *CDKN2A-CDKN2B*. In fact, in previously reported trans-ancestry GWAS meta-analyses of 180 834 T2D cases and 1 159 055 controls from the DIAMANTE Consortium (16), the lead T2D SNV is the same as we report for GDM at *MTNR1B*, *TCF7L2* and *CDKAL1*, and is in strong linkage disequilibrium (LD) at the *CDKN2A-CDKN2B* locus (rs10811661, *r*^2^ = 0.91 across diverse populations in the 1000 Genomes Project (24)). To further investigate the genetic correlation between the two diseases, we extracted GDM association summary statistics from our trans-ancestry meta-analysis for lead SNVs at 222 previously reported loci for T2D from the DIAMANTE Consortium (16) (Supplementary Material, Fig. 1, Table S5). We observed a strong positive correlation in log-ORs for the T2D risk allele between the two diseases: Pearson *r* = 0.573 (*P* < 2.2 × 10^−16^). There was also a highly significant enrichment of GDM associations at T2D loci (50 of 222 lead SNVs with *P* < 0.05 and same direction of effect, binomial test *P* < 2.2 × 10^−16^), indicating that they would be discovered at genome-wide significance with larger effective sample sizes. Indeed, after excluding the four overlapping GDM-T2D loci, a weighted genetic risk score of lead T2D SNVs was significantly associated with GDM (*P* = 9.7 × 10^−123^, pseudo-*R*^2^ = 2.86%). Extending our analyses, genome-wide, using LD-score regression, we observed strong genetic correlation between GDM and T2D: r_G_ (95% CI) 0.744 (0.052, 1.437). Weaker genetic correlations between GDM and other glycaemic traits, BMI and birth weight were also observed (Table 3, Supplementary Material, Table S6). These results are consistent with sharing of genetic determinants of GDM and T2D, although we acknowledge that LD score regression has limited statistical power because of the relatively small GDM sample size, and we note that the correlation from LD-score regression is not bound by −1 to 1, particularly when power is low.

**Figure 1:**
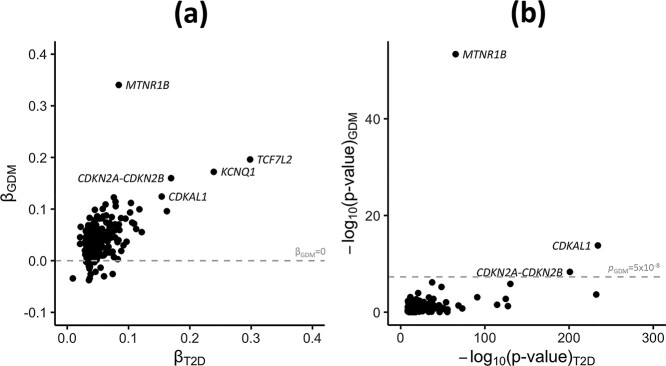
Correlation between GDM and T2D association summary statistics for lead SNVs at previously reported loci for T2D susceptibility. Association summary statistics for GDM were obtained from multi-ancestry GWAS meta-analyses of 5485 cases and 347 856 controls. Association summary statistics for T2D were obtained from multi-ancestry GWAS meta-analyses of 180 834 cases and 1 159 055 controls from the DIAMANTE Consortium. (**a**) Allelic effect sizes (log-ORs) for each disease, aligned to the T2D risk allele, from fixed-effects meta-analysis. The grey line represents log-OR of zero for GDM. (**b**) Association evidence (−log_10_*P*-values) for each disease from meta-regression. The grey line represents genome-wide significance (*P* < 5 × 10^−8^) for GDM. The lead SNV at the *TCF7L2* locus has been removed for ease of presentation (Supplementary Material, Table S4).

**Table 3:** Genetic correlation from LD-score regression of GDM with T2D and glycaemic traits, BMI and birth weight

**Trait**	**Genetic correlation r**_**G**_ **(95% CI)** ^**a**^
T2D	0.744 (0.052, 1.437)
Fasting glucose	0.218 (−0.211, 0.648)
Fasting insulin	0.410 (−0.114, 0.934)
Fasting proinsulin	0.336 (−0.321, 0.993)
2 h glucose (adjusted for BMI)	0.444 (−0.371, 1.260)
HbA1c	0.387 (−0.218, 0.991)
HOMA-B	−0.005 (−0.551, 0.541)
HOMA-IR	0.236 (−0.382, 0.854)
BMI	0.405 (0.001, 0.809)
Birthweight (maternal)	−0.085 (−0.358, 0.189)
Birthweight (fetal)	−0.059 (−0.295, 0.178)

CI: Confidence Interval. T2D: type 2 diabetes. HbA1c: glycated haemoglobin. BMI: body mass index. HOMA-B: homeostasis model assessment of β-cell activity; HOMA-IR: homeostasis model assessment of insulin resistance.

^a^Genetic correlation obtained from LD-score regression is not bound by −1 to 1 and estimates can therefore be found outside these limits due to high imprecision caused by factors such as low sample size in the association summary statistics used.

The most obvious difference in allelic effect sizes between GDM and T2D was observed at the *MTNR1B* locus (Fig. 1). The lead SNV, rs10830963, is the same for both diseases, but the allelic effect on GDM is substantially greater than on T2D: OR (95% CI) for GDM is 1.41 (1.35–1.47) and for T2D is just 1.09 (1.08–1.10). The *MTNR1B* lead SNV is associated, at genome-wide significance, with fasting glycaemic traits in non-diabetic individuals from the Meta-Analysis of Glucose and Insulin-related traits Consortium (MAGIC) Investigators (28,29). The GDM risk allele at the lead SNV is also associated with higher fasting plasma glucose and 1-hour plasma glucose in pregnant women from the Hyperglycemia and Adverse Pregnancy Outcomes (HAPO) Study (30). This SNV also has the strongest association of the maternal glucose-raising allele with higher offspring birth weight in women from the Early Growth Genetics Consortium (31), in line with the known effects of maternal hyperglycaemia on fetal growth. In non-diabetic individuals from the MAGIC Investigators (32), the *MTNR1B* lead SNV has a much larger impact on fasting glucose than those at *TCF7L2*, *CDKAL1* and *CDKN2A-CDKN2B* (33) (Supplementary Material, Table S7). Therefore, the difference in allelic effect sizes between GDM and T2D at *MTNR1B* may reflect the fact that thresholds of fasting plasma glucose used to diagnose GDM are lower than those used to diagnose T2D, meaning that a larger proportion of GDM than T2D cases will have higher fasting glucose that is regulated within the normal range.

To gain insight into the molecular processes and tissues through which GDM association signals are mediated, genome-wide, we then undertook fGWAS enrichment analyses within three categories of functional and regulatory annotations: (i) genic regions (34); (ii) chromatin immuno-precipitation sequence (ChIP-seq) binding sites for 165 transcription factors (35,36) and (iii) 13 unique and recurrent chromatin states in four diabetes-relevant tissues (pancreatic islets, liver, adipose, and skeletal muscle) (37). We observed significant joint enrichment (*P* < 0.05) for GDM associations mapping to protein coding exons, binding sites for FOXA2, NFE2 and TFAP2, and chromatin states in adipose tissue and skeletal muscle that mark enhancers and transcribed regions (Supplementary Material, Table S8). FOXA2 is a pioneer factor involved in pancreatic and hepatic development, and T2D association signals have been previously reported to be enriched for FOXA2-binding sites (38). Skeletal muscle is the most prominent site of insulin-mediated glucose uptake in humans, and enhancers in skeletal muscle have been reported to overlap association signals for metabolic disorders, including T2D, insulin resistance and obesity (39). These enrichment analyses highlight molecular processes and tissues that are broadly consistent with those important in mediating T2D association signals (16), although the involvement of pancreatic islets appears to be less prominent for GDM.

In contrast to the other GDM loci reported in this investigation, the lead SNV at the *HKDC1* locus (rs9663238) demonstrates only weak statistical evidence of T2D association in previously reported trans-ancestry GWAS meta-analyses from the DIAMANTE Consortium (16) (*P* = 0.0083, compared with *P* < 10^−65^ at the other four loci). GDM risk alleles at the lead SNV, and/or at variants in strong LD (European ancestry *r*^2^ > 0.9) with it, have been previously associated, at genome-wide significance, with higher 2-h plasma glucose (2HPG) in pregnant women in the HAPO Study and two replication studies of European ancestry (30), as well as with higher birth weight of first child (likely via greater maternal glucose availability), higher own birth weight (fetal effect independent of the maternal effect on birth weight) and comparative height and body size at age 10 in UK Biobank (40,41) (Supplementary Material, Table S9). The lead SNV is also associated, more strongly in women than men, with higher alanine aminotransferase (ALT) levels in UK Biobank (42). Elevated ALT levels in early pregnancy have been associated with the risk of subsequent development of GDM (43) and genome-wide, we observed positive genetic correlation between the two traits: r_G_ (95% CI) 0.149 (0.005, 0.292). In addition to demonstrating the association of the maternal SNVs at this locus with GDM in the current study, we observed that 99% credible set variants are lead SNVs for *HKDC1* expression quantitative trait loci in a range of tissues in the GTEx Project (44), including visceral adipose, subcutaneous adipose and pancreas (Supplementary Material, Table S10). *HKDC1* (Hexokinase Domain Containing 1) catalyses the phosphorylation of hexose to hexose 6-phosphate and is involved in glucose homeostasis and hepatic lipid accumulation. Haplotypes of variants associated with 2HPG in pregnancy disrupt regulatory element activity and reduce *HKDC1* expression across diverse tissues (including metabolically relevant liver stellate cells and pancreatic islet beta cells), which has been demonstrated to reduce hexokinase activity in multiple cellular models (45). Knockout of hepatic HKDC1 in pregnant mice has also been demonstrated to significantly impair glucose tolerance, highlighting the importance of liver HKDC1 on glucose metabolism during pregnancy (46). Taken together, the evidence from our study and others suggests a more important role for *HKDC1* in glucose metabolism during pregnancy than outside of pregnancy, in addition to independent maternal and offspring effects on early growth, and highlights that while GDM shares many similarities with T2D, there are differences in at least one underlying pathway.

**Figure 2:**
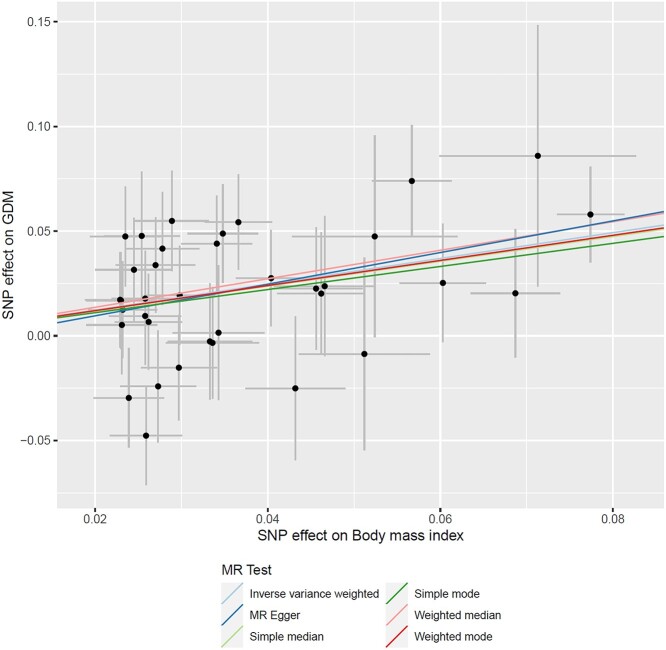
Effects of BMI on GDM from MR analyses. Each point corresponds to an independent SNV (genetic instrument), plotted according to the effect on BMI (on the *x*-axis) and the effect on GDM (log-OR, on the *y*-axis). Horizontal and vertical bars represent the standard errors of effect estimates. The coloured regression lines represent the effect of BMI on GDM from six MR models.

Finally, we used two-sample MR to investigate causal effects on GDM of 282 metabolic measures and risk factors available in the MR-Base GWAS catalogue (www.mrbase.org) (47), including metabolites, anthropometric measures, hormones, immune system phenotypes, kidney traits and metals (Supplementary Material, Table S11). We did not consider glycaemic traits (including HbA1c) because they are used to define GDM status. For each metabolic measure, we selected independent SNVs attaining genome-wide significance with the trait as instrumental variables. For each SNV, we extracted association summary statistics for GDM from the European ancestry-specific meta-analysis because we assessed independence of genetic instruments using LD from European ancestry haplotypes from the 1000 Genomes Project (24). Of the 282 exposures considered, only BMI demonstrated significant evidence for a causal effect on GDM risk at a false discovery rate of 5% (Supplementary Material, Table S11). The estimated causal effect of higher BMI on higher GDM risk was directionally consistent across multiple MR models (Fig. 2). The causal relationship of BMI with GDM is consistent with its effect on T2D (48).

## Discussion

We have conducted the largest and most ancestrally diverse GWAS meta-analysis for GDM, where we identified associations mapping to *MTNR1B*, *TCF7L2*, *CDKAL1*, *CDKN2A-CDKN2B* and *HKDC1*. Our results demonstrated strong correlation in the effects of previously reported associations for T2D and those observed for GDM, and highlighted overlapping molecular mechanisms and tissues that mediate associations for both diseases. In contrast, variation at the *HKDC1* locus is not strongly associated with T2D, but instead plays a more important role in glucose metabolism during pregnancy than outside of pregnancy. The genetic diversity of GWAS contributing to our meta-analysis enabled identification of ancestry-correlated heterogeneity in allelic effects on GDM at two loci. Such heterogeneity could reflect variable impact of different pathophysiology driving glycaemic dysregulation in pregnancy between ancestries and emphasizes the need for increased sample sizes in under-represented population groups. In contrast, results were consistent between GWAS in which all women had a universal blood-based test and those that did not, suggesting little impact from misclassification due to selective use of diagnostic tests only in those deemed to be at high-risk. Finally, MR analyses revealed a significant causal effect of higher BMI on GDM risk, consistent with the causal association observed with T2D. Taken together, these results provide further support for the hypothesis that T2D and GDM are part of the same underlying pathology. However, they also highlight there are pathways to GDM that impact on glucose regulation only in pregnancy, and that additional GDM-specific associations will be revealed through GWAS in larger sample sizes.

## Materials and Methods

### Ethics statement

All human research was approved by the relevant institutional review boards and conducted according to the Declaration of Helsinki. All participants provided written informed consent.

### Study-level analyses

Individuals were assayed with a range of GWAS genotyping arrays, with sample and SNV quality control undertaken within each study (Supplementary Material, Tables S2 and S3). Samples were pre-phased and imputed up to reference panels from the 1000 Genomes Project (phase 1, March 2012 release; phase 3, October 2014 release) (24,49), Haplotype Reference Consortium (25) or population-specific whole-genome sequencing (50–52) (Supplementary Material, Table S3). SNVs with poor imputation quality (*r*^2^ < 0.3 or info < 0.4) and/or minor allele count < 5 were excluded from downstream association analyses (Supplementary Material, Table S3). Association with GDM was evaluated in a regression framework, under an additive model in the dosage of the minor allele, with adjustment for principal components and other study-specific covariates to minimize the population stratification effects (Supplementary Material, Table S3). Phenotype definition and covariate adjustments were not harmonized between GWAS because of differences in individual study design and availability of non-genetic risk factor information. Analyses accounted for structure (population stratification and/or familial relationships) by: (i) excluding related samples and adjustment for principal components derived from a genetic relatedness matrix (GRM) as additional covariates in the regression model; or (ii) incorporating a random effect for the GRM in a mixed model (Supplementary Material, Table S3). Allelic effects and corresponding standard errors that were estimated from a linear (mixed) model were converted to the log-odds scale (53). Study-level association summary statistics (*P*-values and standard error of allelic effects) were corrected for residual structure by means of genomic control (54) if the inflation factor was > 1 (Supplementary Material, Table S3).

### Multi-ancestry meta-analyses

To account for the different reference panels used for imputation across GWAS, we restricted our analyses to autosomal bi-allelic SNVs from the 1000 Genomes Project reference panel (phase 3, October 2014 release) (24) that are also present in the Haplotype Reference Consortium reference panel (25). We considered only those SNVs with MAF > 0.5% in haplotypes in at least one of the five ancestry groups represented in the 1000 Genomes Project (phase 3, October 2014 release).

Our primary multi-ancestry analysis utilized meta-regression, implemented in the MR-MEGA software, which allows for allelic effect heterogeneity between GWAS that is correlated with ancestry (27). We first constructed a distance matrix of mean effect allele frequency differences between each pair of GWAS across a subset of SNVs reported in all studies. We implemented multi-dimensional scaling of the distance matrix to obtain three principal components that defined axes of genetic variation to separate GWAS from the five ancestry groups (Supplementary Material, Fig. S1). For each SNV, we then modelled allelic log-ORs across GWAS in a linear regression framework, weighted by the inverse of the variance of the effect estimates, incorporating the three axes of genetic variation as covariates. Under this model, we tested for association with GDM allowing for allelic effect heterogeneity between GWAS that is correlated with ancestry. We corrected the meta-regression association *P*-values for inflation due to residual structure between GWAS using genomic control adjustment. We considered only those SNVs reported ≥50% of the total effective sample size in downstream analyses.

For each SNV, we also conducted fixed-effects meta-analysis across GWAS under an inverse-variance weighting of allelic log-ORs using GWAMA (55). We corrected standard errors of the resulting effect estimates for inflation due to residual structure between GWAS by genomic control adjustment.

### Defining GDM loci

We identified lead SNVs attaining genome-wide significant evidence of association (*P* < 5 × 10^−8^) in the multi-ancestry meta-regression that were separated by at least 500 kb. Loci were defined by the genomic interval mapping 500 kb up- and downstream of each lead SNV.

### Assessing evidence for multiple distinct association signals at GDM loci

Each GWAS was first assigned to one of the ancestry groups (Supplementary Material, Table S2) represented in the 1000 Genomes Project reference panel (phase 3, October 2014 release) (24). Haplotypes in the panel that were specific to that ancestry group were used as a reference for LD between SNVs across loci for the GWAS in approximate conditional analyses implemented in GCTA (56). For each locus, we applied GCTA to each GWAS to condition on the lead SNV at the locus, using the study-level association summary statistics and matched LD reference. Allelic log-ORs from the approximate conditional analyses across GWAS were modelled in the multi-ancestry meta-regression framework implemented in MR-MEGA (27), incorporating the three axes of genetic variation as covariates, and weighted by the inverse of the variance of the effect estimates. The meta-regression association *P*-values were corrected for inflation due to residual structure between GWAS by using the same genomic control adjustment as in the unconditional analysis. If no SNVs attained genome-wide significant (*P* < 5 × 10^−8^) evidence of residual GDM association in the meta-regression, we concluded that there was a single association signal at the locus.

### Ancestry-specific meta-analyses

We aggregated association summary statistics across GWAS from the same ancestry group via fixed-effects meta-analysis based on inverse-variance weighting of allelic log-OR to obtain effect size estimates using GWAMA (55). We corrected association *P*-values and standard errors of allelic effects from each ancestry group for residual inflation due to structure between GWAS by genomic control adjustment if the inflation factor was > 1. We estimated the mean effect allele frequency across GWAS from each ancestry group, weighted by the effective sample size of the study.

### Investigating the source of heterogeneity in allelic effects on GDM

We extended the meta-regression model implemented in the MR-MEGA software to investigate the impact of ancestry and the use of a universal blood-based test to define GDM status on heterogeneity in allelic effects on GDM at lead SNVs. We modelled allelic log-ORs across GWAS in a linear regression framework, weighted by the inverse of the variance of the effect estimates, incorporating a covariate indicating whether GDM status was defined by a universal blood-based test (Supplementary Material, Table S1) in addition to the three axes of genetic variation.

### Genetic risk score of T2D on GDM

We considered lead SNVs at 237 previously reported loci for T2D from the DIAMANTE Consortium (16) obtained from a multi-ancestry meta-analysis of 180 834 cases and 1 159 055 controls (48.9% non-European ancestry). For each of the 222 SNVs that were reported in our multi-ancestry meta-analysis, we compared association summary statistics (risk allele, other allele, log-OR and *P*-value) for GDM and those reported for T2D. We excluded lead SNVs for T2D that also attained genome-wide significance for GDM. For the remaining SNVs, we regressed the log-ORs for GDM (weighted by their corresponding variances) on the log-OR for T2D, as implemented in grs. summary function (57) of the gtx package in R version 3.4.2. We estimated the percentage of GDM variance explained, as measured by pseudo *R*^2^.

### Genetic correlation between GDM and glycaemic traits

We used LD Hub (58) to perform LD score regression (59) of the European ancestry association summary statistics for GDM on other glycaemic traits. We included T2D (33), fasting glucose (60), fasting insulin (60), fasting proinsulin (60), glucose 2 h post oral glucose tolerance test (adjusted for BMI) (61), HbA1c (62), HOMA-B (63), HOMA-IR (63), BMI (64), birth weight (41) and alanine aminotransferase (42). European ancestry association summary statistics for GDM were filtered so that only SNVs with minor allele frequency >0.01 was included before performing the LD score regression. Genetic correlations between the different glycaemic traits were obtained from the LD Hub lookup centre. Visualization was performed using the R package ggplot2 (65) in R version 3.6.1.

### Enrichment of GDM association signals in genomic annotations

We mapped each SNV across the genome to three categories of functional and regulatory annotations. First, we considered genic regions, as defined by the GENCODE Project (34), including protein-coding exons, and 3′ and 5′ UTRs as different annotations. Second, we considered chromatin immuno-precipitation sequence (ChIP-seq) binding sites for 165 transcription factors: 161 proteins from the ENCODE Project (35) and four additional factors assayed in primary pancreatic islets (36). Third, we considered 13 unique and recurrent chromatin states, including promoter, enhancer, transcribed, and repressed regions, in four diabetes-relevant tissues (37): pancreatic islets, liver, adipose and skeletal muscle. This resulted in a total of 220 genomic annotations for enrichment analyses.

We tested for genome-wide enrichment of GDM associations that map to genomic annotations using fGWAS (66). To do this, we approximated the Bayes’ factor in favour of GDM association for the $j$th SNV by${\varLambda}_j=\exp \Big[\frac{D_j-4\ln{K}_j}{2}\Big]$,where ${D}_j$ is the deviance across *K_j_* contributing GWAS contributing to the multi-ancestry meta-regression (27). We first considered each annotation separately and identified those with significant enrichment (*P* < 0.05). We then used an iterative approach to identify a joint model of enriched annotations from this set. At each iteration, we dropped the annotation from the joint model that minimized the reduction in the penalized likelihood. We continued until no additional annotations worsened the fit of the joint model at nominal significance (*P* < 0.05). We next used the cross-validation likelihood because the significance of parameter estimates from the penalized likelihood cannot be assessed using standard statistical approaches. For the selected joint model, we identified the penalty that maximized the cross-validation likelihood. Finally, we dropped any annotations from the joint model that resulted in a decrease in the cross-validation likelihood.

### Annotation informed fine-mapping of the *HKDC1* locus

At the *HKDC1* locus, we calculated the posterior probability of driving the GDM association for each SNV under an annotation-informed prior model derived from the globally enriched functional and regulatory annotations identified by fGWAS. Specifically, for the $j$th SNV at the locus, the posterior probability ${\pi}_j\propto{\gamma}_j{\varLambda}_j$, where ${\varLambda}_j$ is the Bayes’ factor in favour of GDM association from the meta-regression, derived above. In this expression, the relative annotation-informed prior for the SNV is given by${\gamma}_j=\exp \Big[{\sum}_k{\hat{\beta}}_k{z}_{jk}\Big]$,where the summation is over the enriched annotations, ${\hat{\beta}}_k$ is the estimated log-fold enrichment of the $k$th annotation from the final joint model and ${z}_{jk}$ is an indicator variable taking the value 1 if the $j$th SNV maps to the $k$th annotation, and 0 otherwise. We derived a 99% credible set (67) for the locus by: (i) ranking all SNVs according to their posterior probability ${\pi}_j$; and (ii) including ranked SNVs until their cumulative posterior probability attained or exceeded 0.99.

We conducted a look-up of 99% credible set variants at the *HKDC1* locus for significant (*q* < 0.05) *cis*-expression quantitative trait loci (eQTLs) across tissues in the GTEx Project (44). We reported only those 99% credible variants that were the lead SNV for the eQTL signal.

### MR assessment of the effects of metabolic traits on GDM risk

We systematically searched the MR-Base GWAS catalogue (https://www.mrbase.org) for metabolic measures. We selected all subcategories of metabolites, which included ‘amino acid’, ‘carbohydrate’, ‘cofactors and vitamins’, ‘energy’, ‘fatty acid’, ‘keto acid’, ‘lipid’, ‘metabolite salt’, ‘metabolites ratio’, ‘NA’, ‘nucleotide’, ‘peptide’, ‘protein’, ‘unknown metabolite’ and ‘xenobiotics’. We also selected the following subcategories of risk factors: ‘anthropometric’, ‘hormone’, ‘immune system’, ‘kidney’ and ‘metal’. We identified European ancestry GWAS in MR-Base for each selected metabolic trait. Where more than one GWAS was available for a trait, we gave preference to women-specific studies with the largest sample sizes and numbers of SNVs. Any GWAS undertaken only in men were excluded.

For each metabolic trait with more than five genetic instruments, we conducted MR analyses using a ‘mixture of experts’ (MoE) machine learning approach (68). This approach maximizes statistical power whilst minimizing the impact of horizontal pleiotropy by combining four instrument selection approaches to 14 different MR models. The four approaches for selecting genetic instruments using MoE were: (i) ‘top hits’ corresponding to independent variants associated at genome-wide significance (*P* < 5 × 10^−8^, *r*^2^ < 0.001 using 1000G CEU as the reference population); (ii) ‘directional filtration’ that removed instruments from ‘top hits’ that are likely to be related to the outcome through reverse causation using Steiger filtering (69); (iii) ‘heterogeneity filtering’ that removed instruments from ‘top hits’ that make a substantial contribution to Cochran’s *Q* statistic (*P* < 0.05); and (iv) combined ‘directional filtration’ and ‘heterogeneity filtering’. The 14 MR models were: seven mean-based methods (inverse variance weighting with fixed effects, IVW random effects, MR-Egger fixed effects, MR-Egger random effects and the three Rucker estimates), three median-based methods (simple, weighted and penalized median estimator) and four mode-based methods (simple and weighted mode, each weighted with or without the assumption of no measurement error in the exposure estimates). The best combination of instrument selection-MR method was identified using a variable predicted by MoE, scaled between 0 and 1, where 1 indicates best performance.

For metabolic traits with five or fewer genetic instruments, the MoE approach could not be applied because many of the MR models require larger numbers of SNVs. For these metabolic traits, we used either the Wald ratio estimate (one SNV) or the inverse-variance weighted estimate (between two and five SNVs).

All analyses were conducted in R version 3.6 using the packages ‘TwoSampleMR’ (version 0.5.4) and ‘MRInstruments’ for the MR analyses and ‘EpiCircos’ (https://github.com/mattlee821/EpiCircos).

## Data Availability

Meta-analysis summary statistics can be downloaded from: https://tools.gi.ut.ee/tools/GENDIP_PervjakovaEtAl2022.txt.gz.

## Supplementary Material

Supplementary_Figures_ddac050

Supplementary_Tables_ddac050
